# Novel Use of a Fibular Strut Allograft with Fibular Head in an Elderly Patient with Proximal Humeral Fracture and Severe Metaphyseal Comminution: An Alternative to Shoulder Arthroplasty

**DOI:** 10.3390/jcm13082200

**Published:** 2024-04-11

**Authors:** Jun-Hyuk Lim, Yeong-Seub Ahn, Sungmin Kim, Myung-Sun Kim

**Affiliations:** 1 Department of Orthopedic Surgery, Chonnam National University Medical School and Hospital, Dong-gu, Gwangju 61469, Republic of Korea; ove03@naver.com (J.-H.L.); kimsum83@gmail.com (S.K.); 2 Department of Orthopedic Surgery, Good Morning General Hospital, Pyeongtaek 17874, Republic of Korea; ysahn84@naver.com

**Keywords:** proximal humerus fracture, metaphyseal comminution, intramedullary fibular strut allograft, shoulder arthroplasty

## Abstract

Treatment of a comminuted proximal humerus fracture (PHF) in elderly patients with severe osteoporosis is challenging, often leading to arthroplasty (such as hemiarthroplasty or reverse shoulder arthroplasty) as the treatment of choice. However, arthroplasty does not always guarantee favorable outcomes. In contrast, the use of intramedullary fibular strut allografts provides additional reduction stability during locking plate fixation; however, to our knowledge, there is limited literature on the use of fibular strut allografts, including the fibular head. Here we aim to report the advantages of using a fibular strut containing the fibular head in severe osteoporotic PHFs. We present the case of an 88-year-old female patient with severe osteoporosis diagnosed with a left PHF accompanied by severe metaphyseal comminution following a fall from a chair. Rather than shoulder arthroplasty, we performed osteosynthesis using a fibular strut allograft containing the fibular head. At the one-year follow-up after surgery, we observed excellent bony union and a favorable functional outcome without major complications, such as reduction loss. The novel use of a fibular strut allograft containing the fibular head could be promising for PHFs with severe metaphyseal comminution, potentially avoiding the need for arthroplasty.

## 1. Introduction

Proximal humerus fractures (PHFs) frequently occur in elderly women with poor bone quality, typically as a result of low-energy mechanisms. The treatment of PHFs is challenging and controversial [1]. With the aging population, the incidence of PHFs in elderly patients is increasing. Conservative treatment can be considered as an option for PHFs, whether non-displaced or with some degree of displacement, taking into account the patient’s age and functional demands [2]. However, complex PHFs often result in poor outcomes with conservative treatment; therefore, surgical treatment is often recommended [3]. The available surgical options, including open reduction internal fixation (ORIF) and shoulder arthroplasty (e.g., hemiarthroplasty or reverse total shoulder arthroplasty), have continuously evolved.

Locking plate fixation for severely comminuted osteoporotic PHFs can lead to various complications. Major complications associated with screw perforation with reduction loss or varus collapse are reported more often in elderly patients because of their poor bone quality [4]. Thus, the importance of medial support is increasingly being recognized [4,5,6]. Several studies have reported the importance of medial supporting screws for providing medial support [5,6], with recent emphasis being placed on the importance of cement augmentation [7,8] and strut bone grafting to avoid major complications [9,10].

Since its initial report by Gardner et al., who first described how the intramedullary fibular strut allograft could support the medial column and facilitate fracture reduction in unstable PHFs [11], numerous studies have highlighted its advantages. These include providing fixation stability in unstable osteoporotic PHFs during locking plate fixation and reducing various fracture-related complications [12,13,14,15,16].

Meanwhile, with technological advancements and an increase in surgical volumes for shoulder arthroplasty, the use of shoulder arthroplasty in complex PHFs has recently increased. Shoulder arthroplasty may be indicated, particularly in patients aged 70 and above, as well as those with a high risk of avascular necrosis (AVN), such as Neer three-part or four-part fractures, head-splitting fractures, and those with pre-existing rotator cuff tears [17]. Recent studies have reported that, in elderly patients with complex PHFs, the outcomes of reverse total shoulder arthroplasty (RTSA) are superior to those of open reduction and internal fixation (ORIF) [18], with a lower reoperation rate observed in RTSA [19,20]. However, shoulder arthroplasty is considered a joint salvage procedure, and, to date, the long-term outcomes of shoulder arthroplasty in elderly patients with complex PHF remain limited [21]. Moreover, patients with severe osteoporosis face an increased risk of periprosthetic fracture during surgery, leading to potential complications, such as early implant failure [22,23]. This can escalate the likelihood of revision surgery, which, given that the majority of patients are elderly, becomes challenging, complex, and significantly diminishes postoperative shoulder function.

This study aimed to present a novel surgical method through a case report that can serve as an alternative to shoulder arthroplasty in patients with severely comminuted osteoporotic PHFs extending into the metaphyseal area. Instead of shoulder arthroplasty, we opted for joint-preserving surgery using locking plate fixation augmented with an intramedullary fibular strut containing the fibular head, considering the patient’s poor bone quality. To the best of our knowledge, there are no reports in the literature regarding the use of fibular strut allografts, including the fibular head, during locking plate fixation of PHFs. We further describe the radiological and functional outcomes of the patient.

## 2. Case Presentation

**Figure 1:**
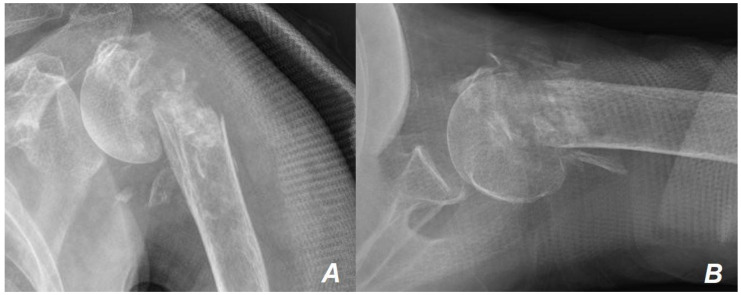
Initial plain radiographs. At the time of the visit, the initial plain radiographs revealed both medial and lateral cortical comminution of the proximal humeral metaphyseal area in the anterior-posterior (**A**) and trans-axillary (**B**) views of the X-ray images.

**Figure 2:**
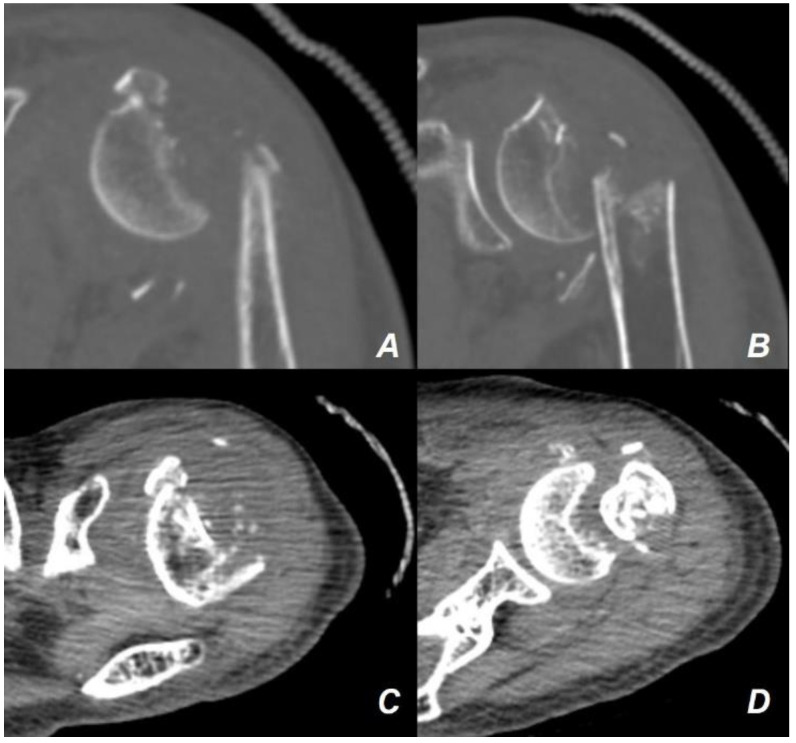
Computed tomographic (CT) slices of the injured arm. The CT slices of the injured arm showed a lesser tuberosity fracture of the proximal humerus, visible in both the coronal (**A**) and axial (**C**) planes. In addition, severe comminutions of the medial and lateral metaphysis areas can be identified in both coronal (**B**) and axial (**D**) planes of the CT slices.

The patient was an 88-year-old woman with left arm pain following a fall from a chair. Plain radiography (Figure 1) and computed tomography (CT) (Figure 2) showed a PHF.

**Figure 3:**
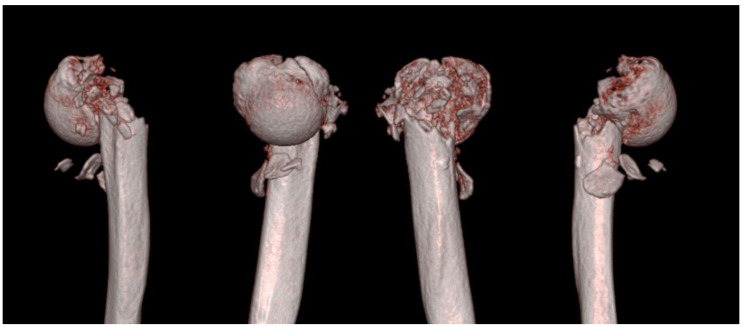
Three-dimensional computed tomography showing the configuration of the patient’s injured arm. The three-dimensional computed tomography showed the severe medial and lateral metaphyseal comminution with varus, flexion, and anteversion of the fracture configuration. A displaced fracture of both the lesser and greater tuberosities was also identified.

The fracture was diagnosed as a comminuted PHF with varus, flexion, and anteversion of the head of the humerus, with severe medial and lateral metaphyseal comminution and displacement of the lesser and greater tuberosities (Figure 3). Evaluation of bone mineral density revealed severe osteoporosis with a T-score of −4.6 at the femoral neck. Despite being 88 years old, the patient had no significant underlying conditions other than severe osteoporosis and mild hypertension controlled with medication. She maintained a functional demand sufficient for independent household activities and daily living (Table 1).

The patient had a previous diagnosis of osteoporosis but had not undergone treatments such as medication. We decided to opt for surgical treatment in this patient for several reasons. First, although the fracture line did not directly involve the head, it presented as an unstable fracture pattern with significant displacement and a large gap between the humeral head and shaft. Second, if left to heal conservatively in its current state, it could result in symptomatic malunion, making functional recovery before surgery unlikely. Third, attempting closed reduction to prevent malunion posed a high risk of additional fractures in other parts of the humerus due to severe osteoporosis, and the patient’s compliance was inadequate to maintain reduction for several weeks, increasing the risk of reduction loss. Lastly, the patient and their caregiver strongly desired surgery.

Shoulder arthroplasty is a viable option for the management of elderly patients, including this patient with an osteoporotic Neer three- or four-part PHF [17]. However, we determined that arthroplasty would be challenging for several reasons, and we could not assure a favorable outcome post-surgery. First, comminution in both the greater and lesser tuberosities complicated tuberosity healing. Second, considering the very low T-score, the patient was expected to experience severe osteoporotic changes in the glenoid, posing challenges for base-plate fixation. Lastly, the possibility of intraoperative periprosthetic fractures during stem insertion was anticipated, which could significantly impact both short-term and long-term outcomes. Instead, we opted for ORIF with locking plate fixation. To prevent major complications such as reduction loss and varus collapse during locking plate fixation, we ensured adequate insertion of medial supporting screws and utilized additional tension-band suture fixation for augmentation. Additionally, we decided to use an intramedullary fibular strut allograft containing the fibular head, offering robust support for both medial and lateral comminution while adequately filling the void defect within the humeral head using the fibular head.

### Surgical Technique

Under general anesthesia, the patient was positioned in the beach chair position. The affected arm was placed on an arm table for easy manipulation and positioning during the procedure. Utilizing a standard deltopectoral approach, a surgical incision of approximately 10–15 cm in length was made just above the coracoid process, tracing along the anterior aspect downward along the beginning of the deltopectoral groove and just above the coracoid process. After identifying the deltopectoral groove and cephalic vein, the pectoralis major and deltoid muscles were located. The deltoid muscle was then retracted laterally, and the pectoralis major muscle was retracted medially. Subsequently, subdeltoid release was performed through finger dissection to create adequate space for plate placement on the lateral side of the proximal humerus.

**Figure 4:**
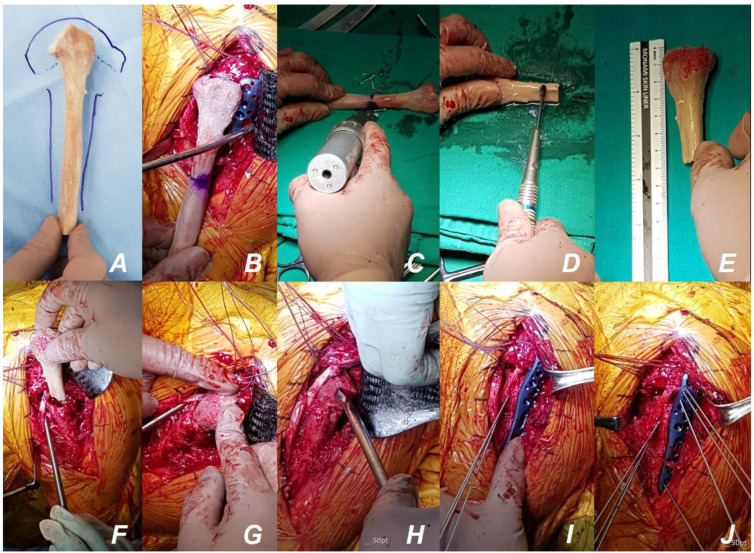
Intraoperative clinical photos of fibular strut allograft preparation. Following the planning of use of the expected configuration with a proximal fibular allograft containing the head (**A**,**B**), the proximal fibular allograft, including the fibular head, was intraoperatively remodeled and decorticated based on the remaining bone configuration (**C**–**E**). The refined fibular strut allograft with the head was inserted into the cavity, where severe comminution with bone loss had developed, through the fracture site after canal preparation (**F**,**G**). The fibular head portion was inserted into the humeral head using a Darrach retractor and an impactor (**H**). After fluoroscopic confirmation of the position of the fibular strut allograft within the proximal humerus, temporary fixation was performed with Kirschner wires (**I**,**J**).

The humeral head and fragments were retracted, and temporary reduction was attempted to ascertain the anatomical configuration. However, due to severe comminution and bone loss in the medial and lateral metaphyseal area, anatomical reduction and maintenance were deemed impossible without supporting the metaphyseal portion. Due to severe osteoporosis, a void defect was identified within the humeral head. To address these challenges, we supported the medial and lateral metaphyseal defects and the void defect of the humeral head with a fresh-frozen fibular strut allograft including the fibular head (Figure 4A).

To ensure optimal fit, we measured the width of the medullary channel of the proximal humeral shaft anteriorly and posteriorly, in addition to the medial and lateral dimensions, before acquiring the fibular strut. The fibular strut, extending from the fibular head to the shaft with sufficient length, was planned to position its metaphyseal area over the main fracture site between the humeral head and shaft (Figure 4A). The distal side of the strut, the shaft portion, was intended to adequately fill the medullary channel of the proximal humerus shaft. Given that the head and metaphyseal area of the fibular strut are relatively thick and the fibular shaft is relatively thin (and fits into the medullary channel of the humerus), we procured a fresh-frozen fibular strut with a shaft corresponding to the smaller size among the measured anterior, posterior, medial and lateral medullary channel widths.

**Figure 5:**
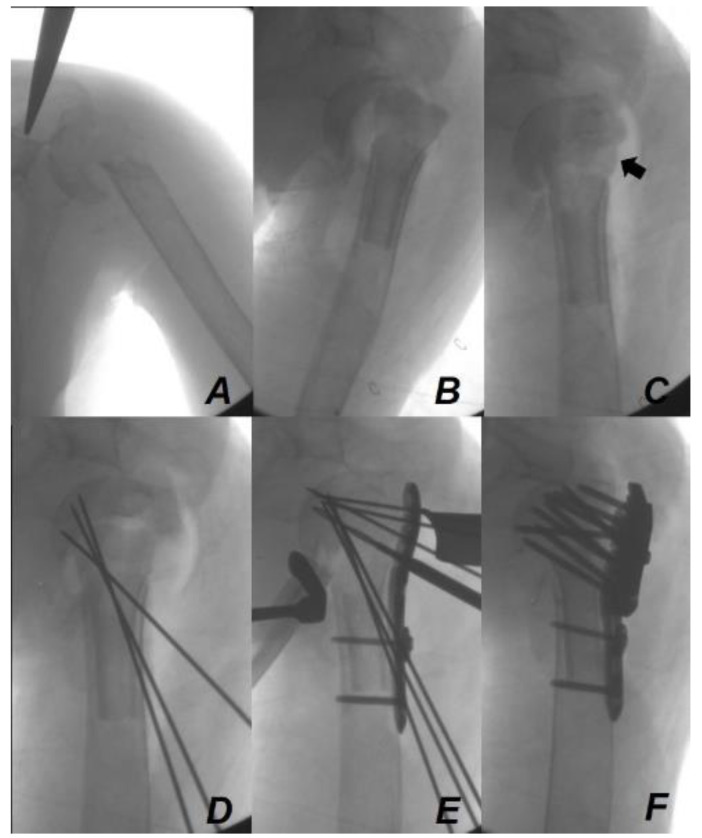
Intraoperative fluoroscopic images of the fixation method using the fibular strut allo-bone-containing head with locking compression plate. After insertion of the proximal fibular strut allograft containing the head (**A**,**B**), humeral head reduction was performed on the allograft (**C**). The defect of the lateral cortex was reconstructed (black arrow) by fibular strut allograft with the head. After confirming via fluoroscopy that the position of the strut bone between the meta-diaphysis and the humeral head was adequate, temporary fixation using Kirschner wires was performed (**D**). While maintaining the reduction state with Kirschner wires, firm fixation was performed using a locking compression plate (**E**,**F**).

**Figure 6:**
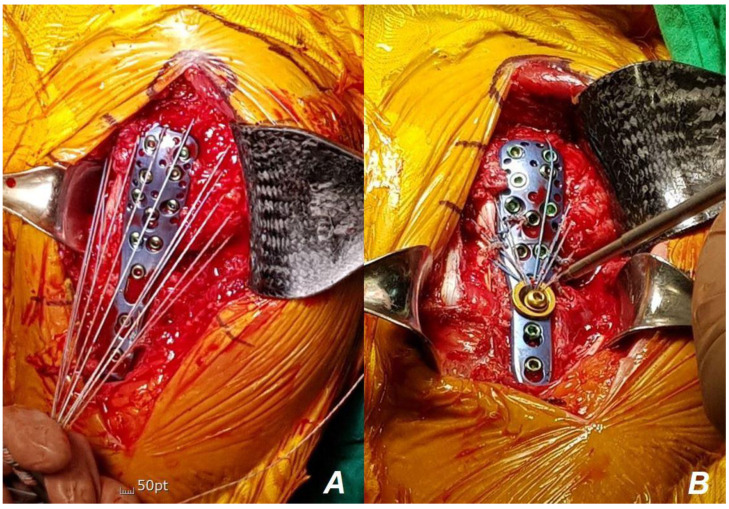
Intraoperative images of locking compression plate application with tension suture fixation. Using non-absorbable suture material, sutures were placed on the subscapularis, supraspinatus, and infraspinatus tendons respectively (**A**). Then, the suture material was passed through two washers. Subsequently, tension was applied to the suture material in the distal direction to its maximum extent, and conventional screw fixation was performed (**B**).

The proximal fibular strut allograft, including the fibular head, was remodeled according to the remaining bony configuration of the patient’s proximal humerus. The length of the fibular strut was determined to sufficiently accommodate the distal part of the locking plate, allowing for the insertion of three or more bi-cortical screws. Additionally, to ensure proper insertion of the shaft portion of the strut into the medullary channel without being too loose or too tight, cortical preparation was performed using an oscillating burr. In our patient, the humerus at the proximal shaft level exhibited a large medullary canal close to a circular shape with a thin cortex. Meanwhile, the shaft of the fibular strut was closer to a triangular shape. During passage through the humerus medulla, there were areas where the edges of the strut caught, necessitating smoothing with a burr (Figure 4B–E). The distal portion of the remodeled fibular strut allograft was initially inserted into the intramedullary canal of the meta-diaphysis through the fracture site (Figure 4F,G). The fibular head was then inserted into the humeral head using a Darrach retractor and an impactor. This allowed for the easy and precise insertion of the proximal portion of the fibular strut allograft into the expected portion of the void defect in the humeral head by sliding down while making contact with the Darrach retractor by pushing the impactor (Figure 4H). Upon ensuring proper positioning of the fibular strut allograft inside the proximal humerus, between the meta-diaphysis and humeral head, and confirming via fluoroscopy, temporary fixation using Kirschner wires was performed (Figure 4I,J). The Proximal Humerus Internal Locking System (PHILOS; DePuy Synthes, Raynham, MA, USA) plates were then used to complete the fixation (Figure 5). Additionally, supplementary tension suture fixation using non-absorbable suture materials with two washers (Figure 6) was performed to enhance stability, thus preventing fixation loss and varus collapse [24,25].

**Figure 7:**
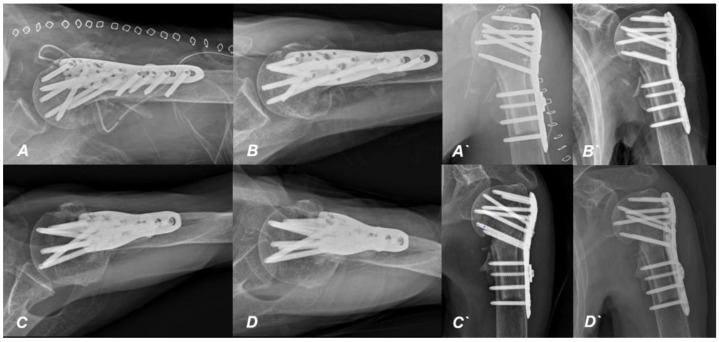
Serial plain radiographic images during the postoperative follow-up period. Continuous radiographic reviews were conducted throughout the outpatient follow-up period following surgery. The images depict radiographs taken at immediate (**A**,**A′**), 3 months (**B**,**B′**), 6 months (**C**,**C′**), and 1 year (**D**,**D′**) postoperatively.

**Figure 8:**
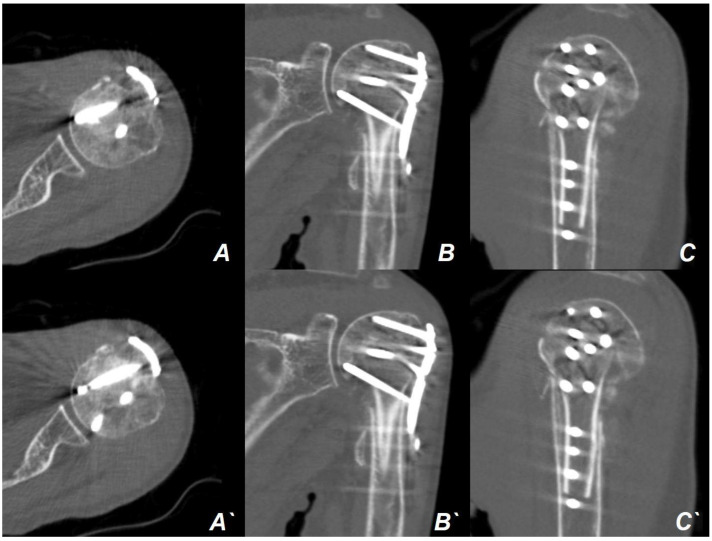
Computed tomography (CT) images performed at the 3-month postoperative follow-up. Progressive bony union was identified in the axial (**A**,**A′**), coronal (**B**,**B′**), and sagittal (**C**,**C′**) slices of the CT images.

**Figure 9:**
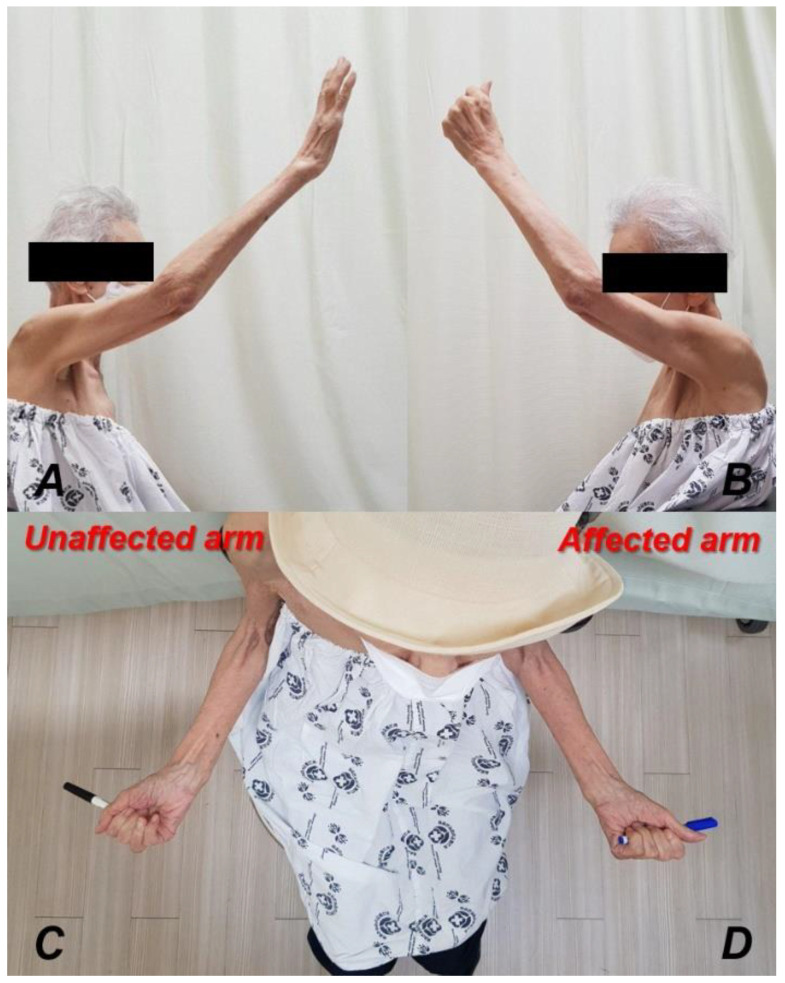
Clinical images of range of motion (ROM) at the last follow-up assessment. The active ROM in the injured arm showed almost the same ROM as that of the unaffected arm, both in forward flexion (**A**,**B**) and external rotation (**C**,**D**).

The affected arm was immobilized in a sling for 2 weeks postoperatively, with gradual passive range of motion (ROM) exercises encouraged thereafter. After 4 weeks, active assisted ROM exercises were performed. To mitigate the risk of periprosthetic fracture due to stress concentration at the distal portion of the plate, the patient and their caregiver were informed during hospital visits not to support themselves by touching the ground when standing up using the affected arm. For osteoporosis treatment after surgery, a combination therapy utilizing parathyroid hormone and denosumab was administered for 1 year post-surgery, followed by a decision to continue lifelong denosumab injections every 6 months thereafter. Subsequent follow-ups were conducted at 2 weeks, 6 weeks, 3 months, 6 months, and 12 months. Additionally, serial plain radiographic images were taken during the postoperative follow-up period (Figure 7). At the 3-month postoperative follow-up assessment, CT scans indicated successful bone union (Figure 8). Active ROM in the affected arm was comparable to that of the unaffected arm (Figure 9). By the 1-year follow-up assessment, favorable functional scoring was observed, with a pain Visual Analog Scale score of 1, Constant−Murley score of 64, University of California at Los Angeles shoulder score of 31, American Shoulder and Elbow Surgeons score of 82, and Disabilities of the Arm, Shoulder, and Hand score of 20.

## 3. Discussion

In our case, we successfully achieved locking plate fixation using an intramedullary fibular strut allograft containing the fibular head as an alternative to shoulder arthroplasty in a patient diagnosed with a severe comminuted PHF extending into the metaphyseal area and complicated by severe osteoporosis.

The patient, being over 70 years old and afflicted with severe osteoporosis, presented with a severely comminuted Neer four-part PHF, potentially indicating shoulder arthroplasty [17]. However, we considered shoulder arthroplasty challenging for several reasons, with concerns regarding achieving favorable functional outcomes in the future. First, the patient’s diagnosis of severe osteoporosis, with a femoral neck T-score of −4.6 on bone mineral density, indicated poor glenoid bone quality. Tabarestani et al. have reported a significant decrease in glenoid bone mineral density as the T-score of the femoral neck decreases [22]. Poor bone quality can impact glenoid fixation during reverse shoulder arthroplasty when implanting the glenoid component, thereby increasing the risk of periprosthetic fracture [23,26]. Secondly, the patient exhibited significant comminution and displacement in both the greater and lesser tuberosities, leading us to anticipate challenges in ensuring proper healing. Several studies have reported that tuberosity healing is essential for successful outcomes in procedures such as hemiarthroplasty, and, although not as critical as in hemiarthroplasty, it remains important for future shoulder function in reverse total-shoulder arthroplasty [27,28,29,30,31].

For the reasons mentioned above, we decided to prioritize ORIF with a locking plate for this patient. In our case, we used an intramedullary fibular strut allograft during locking plate fixation to prevent fixation failure. The objective was to achieve optimal anatomical restoration and maintenance of the medial calcar to prevent varus collapse. Several studies have reported that restoring the medial calcar and avoiding varus alignment during locking plate fixation of PHF are the most crucial factors for successful outcomes of locking plate fixation [4,32]. Moreover, elderly patients with osteoporosis or medial column comminution are prone to increased rates of major complications, such as varus collapse and higher re-operation rates [4,33,34]. With advancements in surgical techniques, Gardner et al. [11] first reported using screws to position the fibular strut allograft more medially to improve medial support and maintain fracture fixation stability.

The patient in this case had sever” ost’oporosis and severe comminution around the surgical neck of the humerus, as well as in the medial and lateral cortices. Furthermore, severe osteoporosis resulted in significant hollowing of the humeral head, with minimal subchondral bone remaining; therefore, we used an intramedullary fibular strut allograft containing the fibular head. Each component of the fibular strut served a specific function and has significance. The head of the strut fills the void defect in the humeral head and assists in securely anchoring the locking screw. This approach is consistent with recent studies reporting the advantage of the fibular strut itself in providing vertical support to the humeral head [35]. The metaphyseal area of the strut provides mechanical support to both the medial and lateral columns at the fracture site with comminution. This may allow the thicker metaphyseal area, unlike the shaft of the strut, to contribute more effectively to the stability of the fracture site in both medial and lateral unstable comminuted PHFs, such as in our patient’s case. Recent biomechanical studies have demonstrated that fibular strut augmentation during locking plate fixation enhances varus stiffness, torsional stiffness, and maximum load failure [36].

We aimed to ensure a precise fit of the fibular strut within the medullary canal of the humerus. This was achieved by meticulously measuring the dimensions of the canal using preoperative CT axial cuts and procuring a strut that closely aligned with our planned specifications prior to purchase. In general, for upper limb fractures including PHFs, it is recommended to achieve fixation at the distal aspect of the fracture involving at least six cortices. Therefore, we determined the length of the strut to encompass all regions where a minimum of three bi-cortical screws could be fixed for adequate fixation. This approach offers the advantage that the locking screw can be inserted through the sturdy portion of the fibular strut, resulting in a stronger purchase. Although not proven by biomechanical studies, one study reported that the use of a fibular strut reduces the force arm of locking screws, thereby decreasing the possibility of screw breakage [35].

We encountered no technical difficulties during surgery as we obtained a fibular strut of the expected size through preoperative planning. In case of size-related errors during the procedure, the advantage lies in the ability to easily resolve the situation through burring, allowing for a straightforward surgical procedure. Salzman et al. recommended the use of additional structural graft when the void defect of the humeral head is sufficiently large enough to make impaction of the strut shaft difficult. Additionally, they suggested contouring the distal portion of the fibular strut using an oscillating saw to ensure stable placement of the strut at the fracture site [37]. Another study reported a technique in which the fibular strut shaft can be placed in the desired position using a K-wire guidewire, which is then used to temporarily hold the fibular strut in place during plate fixation [12]. However, this method may pose technical challenges, as there is a risk of the strut being damaged during K-wire guidewire fixation or slipping into the medullary canal. On the contrary, our fibular strut has a sufficiently large fibular head size, minimizing the need for additional grafting. It provides stable support to the head and is large enough to cover the entire medulla. Once successful grafting is achieved, the surgeon can focus solely on locking plate fixation, offering a technical advantage.

However, several considerations should be taken into account when using fibular strut allografts. First, it does not prevent the risk of AVN in the humeral head, the most significant fracture-related complication that can occur during locking plate fixation. Nonetheless, given our priority of joint-preserving surgery, revision surgery via shoulder arthroplasty can be performed at any time if AVN occurs. This approach preserves bone stock compared to revision arthroplasty due to shoulder arthroplasty failure and makes revision surgery easier. One study reported a mean time of approximately 8.5 months for the detection of global AVN in the humeral head [8]. Fortunately, in up to 1 year post-surgery, AVN of the humeral head has not been detected in our patient. Second, we cannot completely rule out fresh-frozen allograft-related complications such as the transmission of infection or rejection through strut allografting. Lastly, legal restrictions in some countries may result in the unavailability of fibular struts. One study reported that the use of fibular strut allografts was associated with longer surgical times and higher costs compared to groups that did not use fibular struts. There was no significant difference reported in clinical outcomes between the group that used fibular struts and the group that did not [38]. However, this study was limited to two-part and three-part PHFs, and it did not compare the strut allograft group with the shoulder arthroplasty group, indicating its limitations. Most studies commonly describe the advantages of fibular strut augmentation during locking plate fixation in unstable PHFs [9,11,12,13,14,15,16]. Additionally, fibular strut augmentation is cost-saving compared to shoulder arthroplasty.

We recommend that surgeons facing challenging cases of severe comminuted PHFs with severe osteoporosis, where shoulder arthroplasty may be difficult or may not yield favorable outcomes, consider the use of an intramedullary fibular strut allograft containing the fibular head. Our novel surgical method is valuable, as it not only provides structural and volumetric support during locking plate fixation, but also enhances fixation stability, potentially reducing the need for shoulder arthroplasty, facilitating faster bony union and enabling early rehabilitation. However, our study has several limitations. It is a short-term follow-up case report, and the patient had an intact rotator cuff, which may have contributed to achieving a favorable functional outcome separate from bony union issues. Additionally, we used additional techniques, such as tension band suture augmentation, to prevent varus collapse and reduction loss, which could potentially influence the results.

## 4. Conclusions

In conclusion, our novel intramedullary fibular strut allograft, incorporating the fibular head, presents an attractive option for facilitating early bony union and favorable functional outcomes in patients with severe comminuted osteoporotic PHFs undergoing locking plate fixation. It serves as both volumetric support and a strong structural support, providing an alternative to shoulder arthroplasty in challenging scenarios where such an alternative may not be feasible. This approach promotes early bony union and improves functional outcomes for patients.

## Figures and Tables

**Table 1:** Patient demographic data.

Information	Details
**Age at surgery**	88
**Sex**	Female
**Diagnoses**	Severe osteoporosis (T-score −4.6 at the femoral neck)
	Mild hypertension on medication
	Neer 4-part proximal humerus fracture on the left shoulder
**Physical Examination**	Decreased painful range of motion in the left shoulder
**Functional demand**	Independent light household activities
**Osteoporosis medication**	None
**Past medical history**	None

## Data Availability

The data presented in this study are available on request from the corresponding author. The data are not publicly available due to privacy.
